# A clinical study of the hemodynamic and metabolic effects of Zone 3 REBOA for sacral and pelvic tumor resections

**DOI:** 10.1186/s12893-022-01694-w

**Published:** 2022-06-27

**Authors:** Zhiqing Zhao, Jichuan Wang, Taiqiang Yan, Wei Guo, Rongli Yang, Xiaodong Tang, Yi Yang

**Affiliations:** Musculoskeletal Tumor Centre, Peking University People’s Hospital, 11# Xizhimen South Road, Xicheng District, Beijing, 100044 China

**Keywords:** Aortic occlusion, Hemodynamic, Sacropelvic tumor, Hemostasis

## Abstract

**Background:**

Resuscitative endovascular balloon occlusion of the aorta (REBOA) is a key procedure in sacral and pelvic tumor resection that provides hemorrhage control. However, few studies have been performed to capture the effects of REBOA in a nonshock condition and provide a detailed description of the changes occurring with prolonged occlusion time. This study aimed to examine the hemodynamic and metabolic effects of Zone 3 REBOA for sacral and pelvic tumor resections following different periods of REBOA.

**Methods:**

In total, 121 patients who underwent surgical tumor resections of the pelvis and/or the sacrum with the use of aortic balloon occlusion were prospectively enrolled from October 2020 to December 2021. All cases were divided into Group A (occlusion time ≤ 60 min, n = 57) and Group B (occlusion time ≥ 90 min, n = 64). Physiologic parameters were continuously recorded, and laboratory specimens were obtained at regular intervals.

**Results:**

Balloon inflation resulted in a significant increase in SBP from 106 to 120 mmHg and decreased to 96 mmHg immediately following balloon deflation. With the application of REBOA, the median blood loss was only 1200 ml (range, 400–7900). When deflating the REBOA, the arterial pH was lower than baseline (7.36 vs. 7.41, *p* < 0.01), the arterial lactate concentration increased from 0.9 to 1.4 mmol/L (*p* < 0.01), serum potassium measurements increased from 3.99 to 4.12 mmol/L, serum calcium measurements decreased from 2.31 to 2.04 mmol/L, and blood creatinine decreased from 64 to 60 µmol/L. The operating time of Group B was longer than that of patients in Group A, and the patients in Group B needed more blood units to be transfused. Although laboratory measurements, including pH, potassium, calcium, and blood creatinine, were at the same level in two groups comparison, the lactate was significantly higher in Group B after deflation (*p* = 0.01).

**Conclusions:**

The results of this study showed that acceptable hemodynamic and metabolic stability can be attained when the occlusion time of REBOA is more than 90 min, although the long duration of occlusion caused relatively higher lactate levels.

## Introduction

**Fig. 1:**
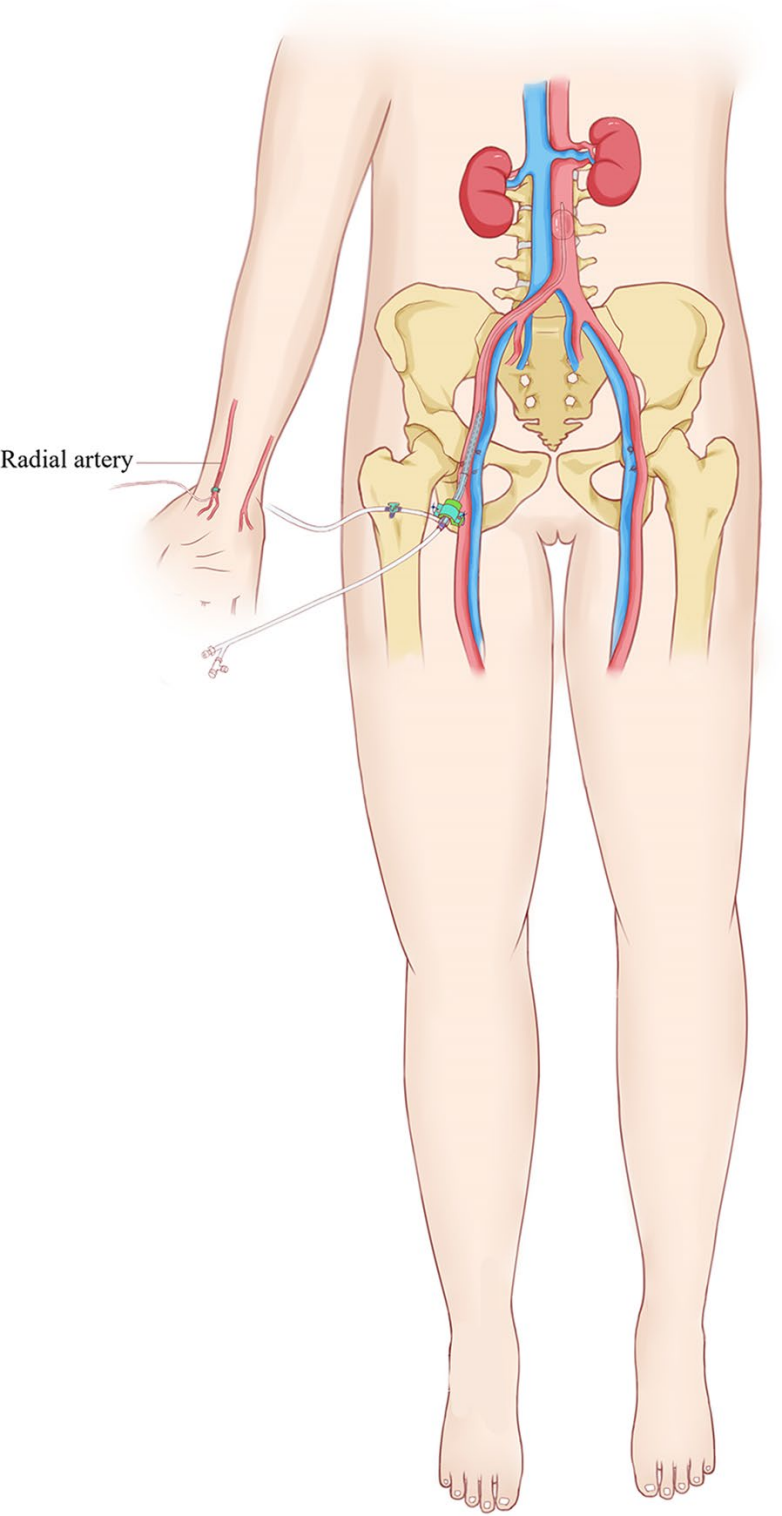
Diagram of infrarenal aorta occlusion (Zone 3 REBOA)

Resuscitative endovascular balloon occlusion of the aorta (REBOA) is a technique that reduces distal blood flow and significantly reduces blood loss. In recent decades, infrarenal aorta occlusion (Zone 3 REBOA) has been successfully used to control bleeding in sacropelvic resections (Fig. 1) [1–7]. However, the prolonged occlusion time can result in artery injury, ischemic necrosis of the distal limb, or multiple organ dysfunction. Some studies [5, 6] have suggested that 60 min is a safe single continuous occlusion duration. Another study [2] showed that a single continuous occlusion duration longer than 90 min caused significant complications, while the overall damage was minor when the occlusion time was less than 90 min. In some animal studies of hemorrhagic shock, occlusion times of 60 or 90 min were associated with substantial ischemia and reperfusion injury (IRI), which increases late mortality [8–12].

To date, there is a paucity of evidence to prove an optimal time duration for aortic occlusion in the setting of Zone 3 REBOA in a nonshock clinical situation. In the present study, we aimed to examine the hemodynamic and metabolic effects of Zone 3 REBOA for sacral and pelvic tumor resections following different occultation duration of REBOA.

## Materials and methods

### Inclusion and exclusion criteria

Patients who had the following characteristics were included in this study: (1) bone and soft tissue tumors of the pelvis and sacrum, (2) age between 18 and 70 years, (3) underwent surgical resections with the use of aortic balloon occlusion, and (4) the infrarenal aorta was occluded only one time during surgery. The contraindications for REBOA were as follows: patients with aortic dissection or aneurysm, renal artery bifurcation caudal to the L2 to L3 disc, history of Stage II hypertension, and manifestation of unstable arterial plaque on abdominal computed tomography (CT).

### Patient characteristics

**Fig. 2:**
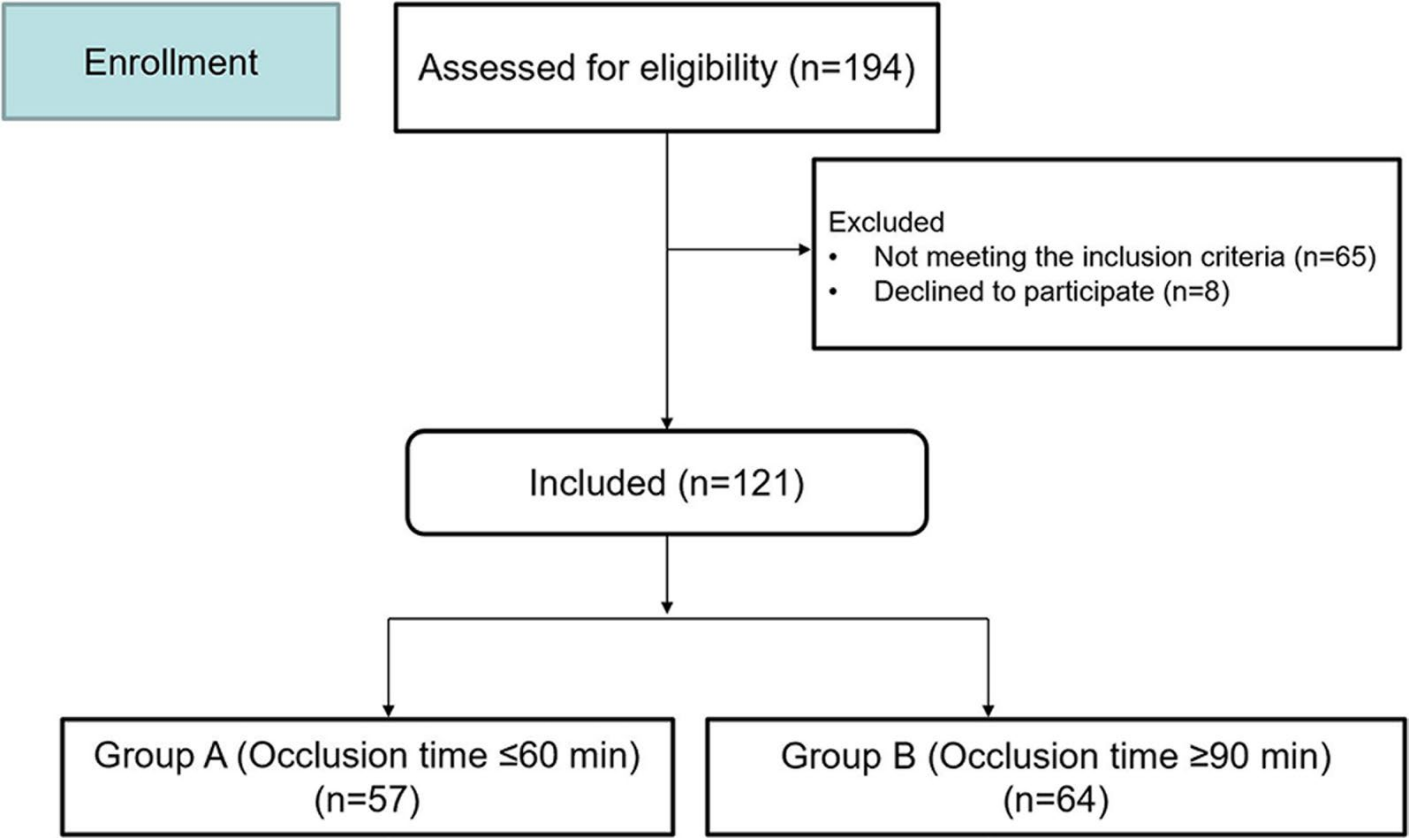
Clinical study design flow diagram

This prospective clinical study performed at a single center was approved by the Ethics Review Committee (ERC) of this institution. In total, 121 patients who underwent surgical tumor resections of the pelvis and/or the sacrum with the use of aortic balloon occlusion were prospectively enrolled in this study from October 2020 to December 2021. The diagnoses consisted of 17 benign tumors and 104 malignant tumors. All cases were divided into Group A (57 patients; occlusion time ≤ 60 min) and Group B (64 patients; occlusion time ≥ 90 min) (Fig. 2).

### Positioning technique of the aortic balloon

Before surgery, a contrast-enhanced abdominal-pelvic CT scan was performed to detect tumor vascularization, to evaluate the diameter of the aorta and femoral artery and to exclude theoretical contraindications to the procedure.

**Fig. 3:**
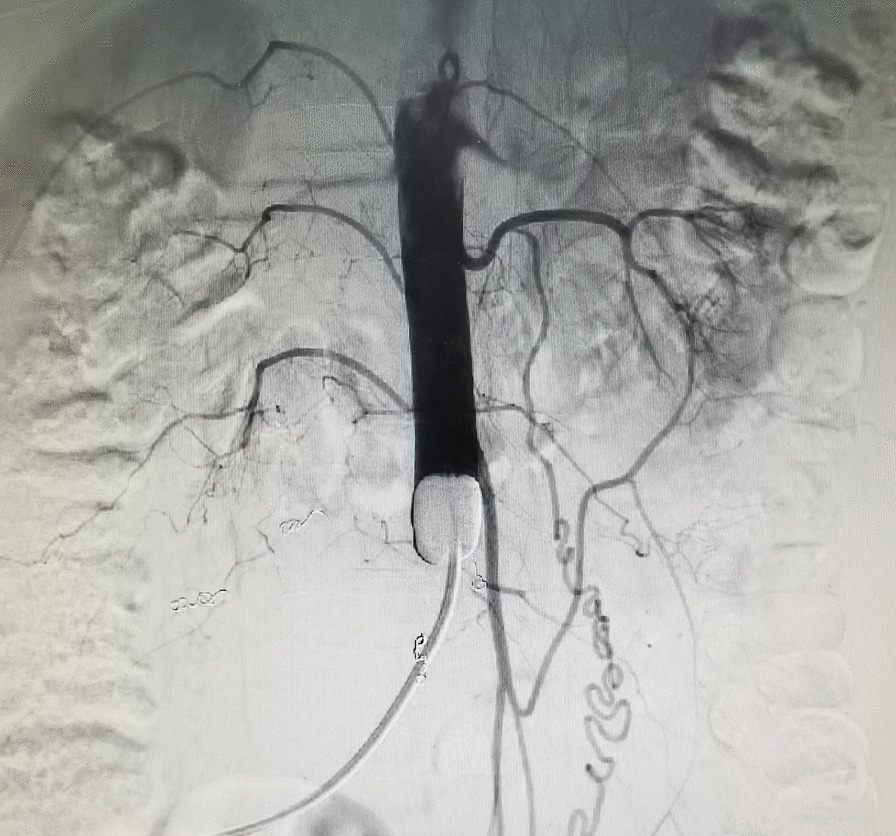
Angiogram should show no blood flow in the distal aorta when the balloon is filled with the injected contrast

After anesthesia induction, the groin were sterilized with iodine solution. The femoral artery contralateral to the tumor in patients with pelvic tumors or the right femoral artery in patients with sacral tumors was punctured. Then, a percutaneous introducer sheath was placed in the femoral artery. Generally, a 10-Fr Coda balloon catheter (Cook Medical, USA) was applied, and it was introduced through an 11-Fr sheath as previously described [4]. In some cases where the aortic diameter was less than 12 mm, a 5.5-Fr balloon catheter (Edward Lifesciences LLC, USA) was used, which is compatible with a 7-Fr introducer sheath. The position of the balloon catheter was confirmed by fluoroscopy utilizing a C-arm (Fig. 3). The distal arterial pressure and urine volume were monitored constantly, which is obligatory and critical to guarantee that the balloon is working properly. Moreover, jugular veins were cannulated to record central venous pressure (CVP), laboratory measurements of blood and medication administration.

In the operating room, the nurse managed the device inflating the balloon with the predetermined volume. Venous blood gas readings, including glucose and electrolytes, were taken at 30 min intervals. The anesthesiologists monitored the intraoperative blood pressure, pulse rate, and hemoglobin values. Every 30 min, 100 ml saline containing 100 units of heparin was injected via the side arm of the femoral sheath catheter to prevent femoral artery thrombosis.

### Data collection

Collection included data pertaining to demographics, location of the tumor, diameter of the abdominal aorta and femoral artery, operating time, occlusion time, total intraoperative blood loss volume, number of blood units per patient (BUPP) transfused, and intraoperative urine volume. Systolic blood pressure (SBP) was recorded just before balloon inflation (baseline) and after aortic occlusion, just following balloon deflation. Before and after occluding, we evaluated blood gas. Laboratory measurements, including hemoglobin (Hb), arterial pondus hydrogenii (pH), lactate, potassium (K^+^), calcium (Ca^2+^), serum creatinine (Scr), prothrombin time (PT), and activated partial thromboplastin time (APTT), were taken at baseline and immediately following balloon deflation.

The intraoperative blood loss was estimated by the sum of the volume of mechanical suction and absorption of dressings and sponges. The need for blood transfusion was determined by assessing the hemodynamic status of the patient.

Balloon-related complication data were collected, such as balloon migration/rupture, aortic rupture, puncture site hemorrhage, aortic aneurysms, acute femoral arterial thrombosis (distal embolus), extremity compartment syndrome, and acute kidney injury.

### Data analysis

Statistical analysis was performed using SPSS version 22.0 (Armonk, NY, USA). Normally distributed data were recorded using the means ± standard deviation (SD), while nonnormally distributed data were presented as the medians and range. Normally distributed data were analyzed using Student’s *t* test, while nonnormally distributed data were analyzed using the Mann–Whitney *U* test. Categorical data were compared by the chi-squared test. The changes in SBP were analyzed using the Friedman test. The differences in laboratory measurements between pre- and post-REBOA inflation were performed by the Wilcoxon signed ranks test. Significance was set at *p* < 0.05.

## Results

Overall, 67 males and 54 females were prospectively included in this study, with a median age of 47 years (range, 18–70) at the time of surgery. Fifty-five percent of lesions were located in the sacrum. The operating time, occlusion time, total intraoperative blood loss volume, number of blood units per patient transfused, and intraoperative urine volume are shown in Table 1.

**Table 1:** The demographics data of 121 patients

Variable	No
Patients	121
Male: Female	67: 54
Median age *(yrs)*	47 (range, 18–70)
Location of the tumor	
Sacrum	67 [55%]
Pelvis	54 [45%]
Diagnosis	
Benign tumor	
Neurofibroma/schwannoma	11
Fibrous dysplasia	3
Hemangioma	3
Malignant Tumor	
Metastatic carcinoma	19
Chordoma	19
Chondrosarcoma	16
Giant cell tumor	14
Osteosarcoma	14
Ewing Sarcoma	4
Malignant fibrous histiocytoma	2
Fibrosarcoma	2
Others	14
Operating Time *(min)*	225 (range, 100–600)
Occulusion Time *(min)*	90 (range, 10–140)
Blood Loss *(ml)*	1200 (range, 400–7900)
Intraoperative blood transfusions *(U)*	7.8 (range, 0–36.4)
Intraoperative urine volume *(ml)*	800 (range, 100–3600)

The mean diameters of the abdominal aorta and femoral artery at the side of puncture were 14.63 mm (SD, 1.96; range, 8.5–19) and 7.45 mm (SD, 1.07; range, 4.5–10.6), respectively. Femoral access was achieved in the angiography room in 47 cases, followed by the operating room (74 patients). The puncture method was blind puncture in all cases. The most frequently used sheaths were 11-Fr (89/121, 73.6%), followed by 7-Fr (32/121, 26.4%).

Balloon inflation resulted in a significant increase in circadian SBP from 106 mmHg (range, 80–155) to 120 mmHg (range, 93–185) and decreased to 96 mmHg (range, 80–120) immediately following balloon deflation (*p* < 0.01). The blood pressure remained stable during the operation.

With the application of REBOA, the median blood loss was only 1200 ml (range, 400–7900), the median operative time was 225 min (range, 100–600), and the occlusion time was 90 min (range, 10–140).

When deflating REBOA, Hb decreased from 126 g/L (range, 83–181) to 111 g/L (range, 72–153) (*p* < 0.01), the arterial pH was lower than baseline (7.36 vs. 7.41, *p* < 0.01), the arterial lactate concentration increased from 0.9 to 1.4 mmol/L (*p* < 0.01), serum potassium measurements increased from 3.99 to 4.12 mmol/L (*p* < 0.01), serum calcium measurements decreased from 2.31 to 2.04 mmol/L (*p* < 0.01), and blood creatinine decreased from 64 to 60 µmol/L (*p* < 0.01) (Table 2).

**Table 2:** Variables recorded just before balloon inflation (baseline), after aortic occlusion, just following balloon deflation

Variables	Baseline	After occlusion	Following balloon deflation	*P* value
Hb *(g/L)*	126 (range, 83–181)	/	111 (range, 72–153)	< 0.01^!^
SBP *(mmHg)*	106 (range, 80–155)	120 (range, 93–185)	96 (range, 80–120)	< 0.01^∮^
CVP *(mmHg)*	5 (range, 1–14)	7 (range, 1–16)	6 (range, 2–17)	< 0.01^∮^
pH	7.41 (range, 7.29–7.57)	/	7.36 (range, 7.25–7.44)	< 0.01^!^
Lactate *(mmol/L)*	0.9 (range, 0.4–1.9)	/	1.4 (range, 0.5–4.1)	< 0.01^!^
K^+^ *(mmol/L)*	3.99 (range, 3.05–4.95)	/	4.12 (range, 2.96–5.24)	< 0.01^!^
Ca^2+^*(mmol/L)*	2.31 (range, 2.03–2.73)	/	2.04 (range, 1.64–2.48)	< 0.01^!^
Scr *(umol/L)*	64 (range,26–116)	/	60 (range, 27–116)	< 0.01^!^
PT *(sec)*	11.7 (range, 10.3–18.9)	/	12.8 (range, 10.1–18.1)	< 0.01^!^
APTT *(sec)*	32 (range, 25–44)	/	29.4 (range, 22.8–50.9)	< 0.01^!^

^!^Wilcoxon signed ranks test

^∮^Friedman Test

### Balloon-related complications

Overall, six patients (6/121, 5%) experienced balloon-related vascular complications. In Group A, one local hematoma at the puncture site and one acute arterial thrombosis (occlusion time was 45 min) occurred. In Group B, there were three local hematomas and one acute arterial thrombosis (occlusion time was 110 min). There was no significant difference in balloon-related vascular complications between the two groups (2/57 vs. 4/64, *p* = 0.78). The patient with limb ischemia was treated with embolectomy, resulting in preservation of the limb. No patient died in the perioperative period.

### Occlusion time ≤ 60 min vs. Occlusion time ≥ 90 min

Comparisons between Group A and Group B were performed. There were no significant differences regarding demography, intraoperative urine volume, or SBP (Table 3). Additionally, there were no differences regarding pH, lactate, blood creatinine, potassium, or calcium at baseline (Table 4). The operating time of Group B was longer than that of the patients in Group A, and the patients in Group B needed more blood units to be transfused. Laboratory measurements, including pH, potassium, calcium, and blood creatinine, were at the same level in Group B compared with Group A after deflation, but the lactate was significantly higher (1.5 vs. 1.3 mmol/L, *p* = 0.01).

**Table 3:** The cmparison between Group A and Group B

Variables	Group A (n = 57)	Group B (n = 64)	*P* value
Male: Female	33: 24	34: 30	0.60*
Age *(yrs)*	48 (range, 18–70)	45 (range, 18–70)	0.53†
Operating time *(min)*	185 (range, 100–500)	260 (range, 180–600)	< 0.01#
Occulusion duration *(min)*	50 (range, 10–60)	100 (range, 90–140)	< 0.01#
Intraoperative blood loss volume *(ml)*	1000 (range, 400–5000)	1350 (range, 400–7900)	0.01#
Intraoperative urine volume *(ml)*	600 (range, 100–3600)	850 (range, 300–2400)	< 0.01#
Intraoperative blood transfusions *(U)*	5.2 (range, 0–26)	7.8 (range, 2.6–36.4)	< 0.01#
Balloon-related complication	2	4	0.78*
SBP at baseline *(mmHg)*	107 (range, 80–140)	105 (range, 90–155)	0.80#
SBP after deflation *(mmHg)*	95 (range, 80–120)	96 (range, 80–119)	0.53#

*Chi squared test

^†^Student’s *t* test

^#^Mann–Whitney *U* test

**Table 4:** The cmparison of laboratory measurements between Group A and Group B

Variables	Group A (n = 57)	Group B (n = 64)	*P* value
Hb at baseline *(g/L)*	129 (range, 89–170)	123 (range, 83–181)	0.02#
Hb after deflation *(g/L)*	115 (range, 81–153)	108 (range, 72–144)	0.02#
pH at baseline	7.41 (range, 7.33–757)	7.41 (range, 7.29–7.48)	0.69#
pH after deflation	7.36 (range, 7.25–7.44)	7.38 (range, 7.26–7.42)	0.17#
Lactate at baseline *(mmol/L)*	0.9 (range, 0.4–1.9)	0.85 (range, 0.5–1.9)	0.97#
Lactate after deflation *(mmol/L)*	1.3 (range, 0.5–3.7)	1.5 (range, 0.7–4.1)	0.01#
K^+^ at baseline *(mmol/L)*	3.94 (range, 3.05–4.58)	3.99 (range, 3.29–4.95)	0.46#
K^+^ after deflation *(mmol/L)*	4.16 (range, 2.96–5.24)	4.11 (range, 3.2–4.95)	0.59#
Ca^2+^ at baseline *(mmol/L)*	2.32 (range, 2.03–2.73)	2.30 (range, 2.03–2.63)	0.39#
Ca^2+^ after deflation *(mmol/L)*	2.05 (range, 1.64–2.29)	1.99 (range, 1.67–2.48)	0.08#
Scr at baseline *(umol/L)*	67 (range, 26–116)	61.5 (range, 34–113)	0.19#
Scr after deflation *(umol/L)*	61 (range, 27–116)	59 (range, 34–99)	0.76#

^#^Mann–Whitney *U* test

## Discussion

The resection of bone and soft tissue tumors in the pelvis and sacrum presents a big challenge due to the large volume of the tumor and the complexity of the anatomical region. This particular anatomy and the rich blood supply of the pelvis and sacrum can lead to significant intraoperative blood loss, up to 10,000 mL [4]. Reducing blood loss during pelvic and sacral tumor resection is critical. An occluding lower abdominal aortic balloon can temporarily inflate the abdominal aorta, thereby reducing distal blood flow during the period of tumor resection. In recent decades, abdominal aortic balloon occlusion has been successfully implemented to control bleeding in sacropelvic resections [3, 4, 7]. To date, multiple clinical studies and animal studies [1, 8, 9, 13] and translational hemorrhagic models have described the efficacy of REBOA in a state of hemorrhagic shock, but few studies have captured the effects of REBOA in a nonshock condition and provided a detailed description of the hemodynamic and metabolic changes occurring with prolonged occlusion time.

Previous studies have reported that patients with aortic balloon occlusion showed a significantly shorter mean operating time, lower blood loss, lower blood transfusion volume, and lower postoperative drainage volume than patients without occlusion[1, 4]. Although the procedure is effective at creating temporary hemorrhage control, it confers risks associated with prolonged occlusion time. The intent of the present study was to prospectively evaluate Zone 3 REBOA in sacral and pelvic tumor resections with regard to hemodynamic and metabolic change. In addition, we compared the effect of more than 90 min of continuous REBOA with that of less than 60 min. These detailed hemodynamic and metabolic findings have not been reported previously to the best of our knowledge.

With the use of REBOA in Zone 3, the median intraoperative blood loss volume was only 1200 ml, and the median operative time was 225 min. The median occlusion time was 90 min (range, 10–140). This procedure assisted the surgeon in achieving the clear surgical margin. In addition, intraoperative contamination was also minimized. The results of this study uphold the current recommendations for lower abdominal aortic balloon occlusion as an effective technique to significantly reduce blood loss and offer a more visible operation field [1, 3–6].

In the current study, the SBP increased significantly from 106 mmHg just before balloon inflation to 120 mmHg after aortic occlusion and decreased to 96 mmHg just following balloon deflation (*p* < 0.01). The observed hemodynamic response is due to the effect of increasing cardiac afterload and blood volume redistribution. Relatively more blood was reserved in the proximal trunk. If the arterial blood pressure increases significantly during aortic occlusion, vasodilators can be used to control it. When we deflated the balloon, the inflammatory response resulted in pooling of the blood in the distal parts of the body, and a dramatic decrease in SBP was caused by reactive hyperemia in the distal body [14]. However, transient hypotension can increase to basal levels after rapid infusion of solution and blood transfusion. Overall, the blood pressure remained stable during the operation.

When deflating REBOA, the hemoglobin level decreased from 126 to 111 g/L (*p* < 0.01), potassium increased from 3.99 to 4.12 mmol/L (*p* < 0.01), lactate increased from 0.9 to 1.4 mmol/L (*p* < 0.01), calcium decreased from 2.31 to 2.04 mmol/L (*p* < 0.01), and blood creatinine decreased from 64 to 60 µmol/L (*p* < 0.01). The reduction in hemoglobin was due to two factors: resection of the tumor results in blood loss, and reperfusion after deflation causes redistribution of blood flow. Ischemia and reperfusion cause reactive hyperemia mediated by the inflammatory response, leading to the pooling of blood in the distal parts of the body, whereby systemic hypovolemia arises [1]. The deflation caused increased pH and potassium concentrations after reperfusion. A plausible explanation could be the ischemic insult caused by a combination of hemorrhagic shock and hypoperfusion due to REBOA. During reperfusion, potassium and ischemic metabolites from necrotic cells are reintroduced into circulating blood. IRI also caused hyperkalemia in all groups through acidosis and reduced excretion in the kidneys. During the procedure, urine output monitoring is important. If the urine output is less than 0.5 ml·kg·h, the position of the balloon may be too high and may require an adjustment. In this study, the median intraoperative urine output was 800 ml (range, 100–3600). Interestingly, serum creatinine was lower than before occlusion due to higher SBP during occlusion and fluid supply. Therefore, acute kidney injury was not observed in this study.

In this study, except for lactate being higher in Group B than that in Group A after deflation (*p* = 0.01), other laboratory measurements, including pH, potassium, calcium, and serum creatinine, were at the same level in this study (*p* > 0.05). Many animal data have suggested that prolonged occlusion of the aorta is associated with IRI, high rates of spinal cord ischemia, and potentially an increased risk of death [13, 15, 16]. Profound distal ischemia means that there is a maximal duration of use for REBOA that cannot be extended. In previous studies, the researchers suggested that a continuous occlusion duration should be better within 60 min. However, in the current study, we performed a Zone 3 balloon occlusion. The occlusion balloon of the lower abdominal aorta was situated distal to the superior mesenteric artery and the renal artery. Therefore, it did not impair the blood supply of the abdominal viscera or kidney.

There were some limitations to this study. First, in the setting of Zone 3 REBOA in a nonshock model, SBP and some laboratory measurements were monitored and controlled by anesthetists. Therefore, the results of more than 90 min of occlusion of the REBOA were different from some previous reported shock models. Second, the study did not detect a change in ionized calcium among laboratory tests. Third, to obtain differential validity between the two groups, we did not include cases whose occlusion time was between 60 and 90 min.

The results of this study showed that acceptable hemodynamic and metabolic stability can be attained when the occlusion time is more than 90 min in a nonshock clinical sitting, although the long duration of occlusion may cause relatively higher lactate levels.

## Data Availability

The datasets used and/or analyzed during the current study are available from the corresponding author on reasonable request.
